# Does fasting in Ramadan ameliorate Lipid profile? A prospective observational study

**DOI:** 10.12669/pjms.304.4690

**Published:** 2014

**Authors:** Arash Akaberi, Alireza Golshan, Maryam Moojdekanloo, Maryam Hashemian

**Affiliations:** 1 Arash Akaberi, Research Center of Natural Products Safety and Medicinal Plants, North Khorasan University of Medical Sciences, Bojnurd, Iran.; 2 Alireza Golshan, Research Center of Natural Products Safety and Medicinal Plants, North Khorasan University of Medical Sciences, Bojnurd, Iran.; 3 Maryam Moojdekanloo, Research Center of Natural Products Safety and Medicinal Plants, North Khorasan University of Medical Sciences, Bojnurd, Iran.; 4 Maryam Hashemian, Research Center for Advanced Medical Technologies, Sabzevar University of Medical Sciences, Sabzevar, Iran.

**Keywords:** Body Mass Index, Fasting, Lipoprotein, Lipid profile, Ramadan, Risk factor

## Abstract

***Background*** ***and Objectives:*** There are conflicting data on the effects of fasting in Ramadan in Muslim countries on Lipid profile. We aimed to evaluate the effect of fasting on lipid profiles and some ratios which are strong for predicting cardiovascular disease.

***Methods:*** This prospective observational study was done in Iran in 2012. Forty three persons were enrolled into the study. Their anthropometric measurement was done. Fasting plasma high density lipoprotein cholesterol (HDL), low density lipoprotein cholesterol (LDL), total cholesterol (TC), and triglyceride (TG) were measured at baseline and after one month fasting during Ramadan by standard methods. Paired t test were used to compare lipid profiles before and after the intervention.

***Results*** ***:*** High density lipoprotein cholesterol was 33.10±6.53 mg/dL at baseline and increased to 42.49±8.44mg/dL (P <0.001). Fasting in Ramadan decreased serum LDL/HDL and TG/HDL ratios significantly (P <0.001). Triglyceride levels were unaffected. Low density lipoprotein and total cholesterol levels increased (P=0.008). Changes did not differ significantly between men and women.

***Conclusion:*** Fasting in Ramadan is effective to ameliorate High density lipoprotein, and LDL/HDL and TG/HDL ratios. Omitting one meal may be considered to control High density lipoprotein level.

## INTRODUCTION

Elevated low-density lipoprotein cholesterol (LDL) and decreased high-density lipoprotein cholesterol (HDL) is known as Dyslipidaemia and is a risk factor for atherosclerosis in coronary artery disease (CAD).^1^ The Framingham study was the first to show that low levels of HDL is a major risk factor for CAD. Following studies have confirmed this claim.^2^ The theory was supported in a meta-analysis, including 302430 subjects from 68 prospective studies.^3^ Therapeutic strategies usually focus on lowering LDL. Since cardiovascular disease are even the cause of about 50% of all deaths^4^, recent researchers try to find options to increase HDL.^2^ But, current drugs resulting in HDL increase are scarce and their effect is conflicting. Therefore, Dietary and lifestyle changes can help increase HDL levels.^5^ One of these changes in diet happens during Ramadan. Muslims refrain from eating and drinking from dawn to sunset in one month of a year called Ramadan. In other words in this month the frequency of food consumptions decrease. Since there is no limitation in religious law in amount or kind of food they eat or drink^6^, It seems that study on the effect of fasting in Ramadan is accordance to decrease in frequency of food consumption. Some Studies in Muslim countries have shown that fasting in Ramadan may improve lipid profile^6^^,^^7^ but there are opposite result in others.^8^^,^^9^ Some studies are limited to special group.^6^^,^^10^ In this study, changes in HDL and other lipid profile have been evaluated in Iran as a Muslim country with different culture and food pattern from previous studies. The healthy effect of fasting in Ramadan on HDL can be generalized not only in Islamic country, but also to all countries thorough decrease in food frequency.

## METHODS

***Study design: ***Forty three healthy persons were recruited into the study. They were between 20 and 40 years old and were not obese [body mass index (kg/m2) range: 18.5–30.0]. The exclusion criteria were pregnancy and being unable to continue fasting more than ten days. The subjects visited the clinical research center of North Khorasan (Bjnord, Iran), one day before Ramadan at baseline and first day of Shaban (next month). They were provided detailed information about the purpose of the study, amount of X-ray they would be exposed and written informed consents were taken one day before Ramadan. Subjects were allowed to eat whatever they liked during non-fast time. The Institutional Review Board of the North Khorasan and the ethic committee approved the study on July 2011 with code 250.

***Data collection: ***After about 12-14 hours of fasting, approximately 4 ml of whole blood was drawn from left arm of each subject, one day before Ramadan at baseline and first day of Shaban. Pregnancy was ruled out. Subjects weighted without shoes and in light clothing with one scale. BMI was calculated as: weight (kg)/height (m2).Hip circumference was measured at the widest level above the trochanters, and waist circumference was measured at the narrowest level. We used one non stretchable tape and the measurement was done in upright position. All measurement was conducted with a trained interviewer.

***Statistical analysis: ***Data were analyzed thorough the Statistical Package for the Social Sciences (SPSS, version 15). Variables in both before and after Ramadan were checked for normality by One-Sample Kolmogorov-Smirnov Test and they had normal distribution. Continuous variables were expressed as mean± SD and were compared using independent t tests, Paired t test. Assuming a power of 90% and α=5%, comparing HDL-C over the month of Ramadan, could be demonstrated with a sample size of 35 patients. A P-value<0.05 was considered to be statistically significant.

## RESULTS

Forty three persons were enrolled into the study (men=51.2% and women=48.8%). The average age was 29.72±4.48 (29.4± 4 in men and 30.2± 5.2 in women, p=0.55). There was no significant difference between men and women in BMI (26.5 2.9 vs. 26.0± 3.8, p=0.607). HDL cholesterol was 33.10±6.53 at baseline and increased to 42.49±8.44 (P <0.001). Total cholesterol increased during Ramadan (p=0.008). LDL cholesterol was 93.21±24.55 at baseline and increased to 98.36±21094 (p=0.003). Triglyceride changes were not significant. Serum LDL/HDL and TG/HDL ratios decreased during one month fasting in Ramadan (Table-I).

This study was conducted during Ramadan in August 2011. The average of fast period was 15 h 49 min. The temperature was similar on all study days, ranging between 21 and 39˚C (mean=31.8 ˚C).

Lipid profile changes did not differ significantly between men and women. There was no smoker in our study. The subjects lost 1.47±1.32 kg of their baseline body weight (P<0.001). The relevance between lipid profile and weight change was not significant. 

## DISCUSSION

We found that Ramadan significantly increased HDL, LDL and total cholesterol levels, whereas it decreased serum LDL/HDL and TG/HDL ratios. LDL/HDL and TG/HDL ratio has been proposed to be a good predictor for cardiovascular disease. Triglyceride levels were unaffected.

**Table-I:** Change in lipid profile in forty three subjects in Ramadan

	***Before*** ***(Mean*** ***±*** ***SD)***	***After*** ***(Mean*** ***±*** ***SD)***	***Changes*** ***(Mean*** ***±*** ***SD)***	***P value***
Total cholesterol(mg/dL)	172.38±37.62	180.23±31.78	7.85±17.45	0.008
HDL(mg/dL)	33.10±6.53	42.49±8.44	9.38±6.23	P <0.001
LDL(mg/dL)	93.21±24.55	98.36±21.94	5.15±10.13	0.003
TG(mg/dL)	113.33±49.74	111.87±59.55	-1.46±31.37	0.773
TG/HDL	3.62±1.79	2.87±1.92	-0.743±1.083	P <0.001
LDL/HDL	2.91±0.85	2.41±0.71	-0.499±0.419	P <0.001

HDL causes the movement of harmful lipids, particularly excess cholesterol, from peripheral tissues, back to the liver for safe disposal. HDL also blocks induced aggregation of LDL enzymatic ally, improves vascular reactivity by vasodilatation thorough inducing nitric oxide production, and inhibits inflammation, chemo taxis and thrombosis. Also facilitates the emigration of macrophages out of the arterial wall.^2^^,^^11^^,^^12^ Increase in HDL level in our study is in agreement with others.^13^^-^^16^ Few studies have reported decrease in HDL levels.^17^ This difference may result from the differences in physical activity levels, because some people decrease their activity during Ramadan, while some increase their praying which is equivalent to moderate physical activity.^7^ Also sleeping habits may be altered and smoking is limited to non fasting time only. Some studies have concluded that the change in lipid profile depends on weight changes.^18^ In the present study HDL increased with weight loss, but the relevance was not significant, it could be related to small sample size. Increasing HDL by fasting or omitting one meal may constitute a non pharmacological method to ameliorate HDL level which cannot be controlled well by drugs at this time. We suggest that future studies evaluate this finding in people with lipid disorders.

In the present study, LDL levels increased during Ramadan fasting. Our findings on the LDL are comparable with some previous findings^17^, which may be related to weight loss during Ramadan fasting. However, some have found no change^15^, or even decreased levels of LDL cholesterol during Ramadan.^13^^,^^14^ This difference may result from the differences in the food habits and the amount and composition of meal consumed by the subjects in various populations. Some people increase their intake of carbohydrate and fat during Ramadan.

In this study total cholesterol levels increased during Ramadan fasting while triglyceride levels were unaffected. Our findings are in contrast to results from Adlouni et al. in Morocco^13^, where a significant reduction in total cholesterol and triglyceride levels was seen. A study in Kuwait showed no significant changes in total cholesterol and triglyceride levels.^19^ These conflicting data could be explained by Mediterranean dietary habits in Morocco, which does not usually exist in countries like Iran and Kuwait.

Also we have reported TG/HDL level in Ramadan for the first time to our knowledge. According to our finding, although TG levels were unaffected, TG/HDL decreased during Ramadan. TG/HDL ratio is a better predictor of coronary artery disease than lipid alone and to be even stronger for predicting myocardial infarction than LDL/HDL ratio.^20^ Because TG/HDL ratio is inversely correlated with large and less dense HDL2 particles, which are protective vs. atherogenic small dense HDL3 particles and also is associated with insulin resistance. Putting together the results of TG and HDL in other research shows that probably in their research from different population TG/HDL has been decreased in most of them and it could be concluded that Ramadan could be protective of coronary artery disease because of its effect on TG/HDL ratio as a better predictor of coronary artery disease. We suggest that future studies report these ratios which are more important than lipid alone.

***Limitations of the study: ***There were several limitations in the present study. (1) We did not collect details on the food items the subjects consumed which might change lipid profile (2) sleeping habits of the subjects were not recorded. 

## CONCLUSION

Fasting in Ramadan is effective to ameliorate HDL, and LDL/HDL and TG/HDL ratios, and could be protective of coronary artery disease. In addition, omitting one meal may be considered to control HDL level even without decreasing calorie intake. Studies with large sample size with control group was warranted to evaluate this finding. Follow up studies are recommended to evaluate the duration of these healthy effect of Ramadan on lipid profile.

## References

[1] Arca M, Montali A, Valiante S, Campagna F, Pigna G, Paoletti V (2007). Usefulness of atherogenic dyslipidemia for predicting cardiovascular risk in patients with angiographically defined coronary artery disease. Am J Cardiol.

[2] Ali KM, Wonnerth A, Huber K, Wojta J (2012). Cardiovascular disease risk reduction by raising HDL cholesterol - Current therapies and future opportunities. British Journal of Pharmacology.

[3] Angelantonio ED, Sarwar N, Perry S, Kaptoge S, Ray K, Thompson A (2009). Major lipids, apolipoproteins, and risk of vascular disease. JAMA.

[4] Greenow K, Pearce NJ, Ramji DP (2005). The key role of apolipoprotein E in atherosclerosis. J Mol Med (Berl).

[5] Hausenloy DJ, Yellon DM (2009). Enhancing cardiovascular disease risk reduction: raising high-density lipoprotein levels. Curr Opin Cardiol.

[6] Saada AD, Attou SG, Belkacemi L, Chabane AO, Italhi M, Bekada AMA (2010). Effect of Ramadan fasting on glucose, glycosylated haemoglobin, insulin, lipids and proteinous concentrations in women with non-insulin dependent diabetes mellitus. African J Biotechnol.

[7] Shehab A, Abdulle A, El Issa A, Al Suwaidi J, Nagelkerke N (2012). Favorable changes in lipid profile: the effects of fasting after ramadan. PLoS One.

[8] Akturk IF, Biyik I, Kocas C, Yalcin AA, Erturk M, Uzun F (2012). Effects of Ramadan Fasting on Lipid Profile, Brain Nadriuretic Peptide, Renal Functions and Electrolyte Levels in Patients with Hypertension. Int J Cardiol.

[9] Khafaji HA, Bener A, Osman M, Al Merri A, Al Suwaidi J (2012). The impact of diurnal fasting during Ramadan on the lipid profile, hs-CRP, and serum leptin in stable cardiac patients. Vasc Health Risk Manag.

[10] Dikensoy E, Balat O, Cebesoy B, Ozkur A, Cicek H, Can G (2009). The effect of Ramadan fasting on maternal serum lipids, cortisol levels and fetal development. Arch Gynecol Obstet.

[11] Williams KJ (2012). What does HDL do? A new mechanism to slow atherogenesis – But a new problem in type 2 diabetes mellitus. Atherosclerosis.

[12] Singh V, Sharma R, Kumar A, Deedwania P (2010). Low high-density lipoprotein cholesterol: current status and future strategies for management. Vasc Health Risk Manag.

[13] Adlouni A, Ghalim N, Benslimane A, Lecerf JM, Saile R (1997). Fasting during Ramadan induces a marked increase in high-density lipoprotein cholesterol and decrease in low-density lipoprotein cholesterol. Ann Nutr Metab.

[14] Fakhrzadeh H, Larijani B, Sanjari M, Baradar-Jalili R, Amini MR (2003). Effect of Ramadan fasting on clinical and biochemical parameters in healthy adults. Ann Saudi Med.

[15] Maislos M, Khamaysi N, Assali A, Abou-Rabiah Y, Zvili I, Shany S (1993). Marked increase in plasma high-density-lipoprotein cholesterol after prolonged fasting during Ramadan. Am J Clin Nutr.

[16] Nematy M, Alinezhad-Namaghi M, Rashed MM, Mozhdehifard M, Sajjadi SS, Akhlaghi S (2012). Effects of Ramadan fasting on cardiovascular risk factors: a prospective observational study. Nutr J.

[17] Ziaee V, Razaei M, Ahmadinejad Z, Shaikh H, Yousefi R, Yarmohammadi L (2006). The changes of metabolic profile and weight during Ramadan fasting. Singapore Med J.

[18] Hallak MH, Nomani MZ (1988). Body weight loss and changes in blood lipid levels in normal men on hypocaloric diets during Ramadan fasting. Am J Clin Nutr.

[19] Akanji AO, Mojiminiyi OA, Abdella N (2000). Beneficial changes in serum apo A-1 and its ratio to apo B and HDL in stable hyperlipidaemic subjects after Ramadan fasting in Kuwait. Eur J Clin Nutr.

[20] Wakabayashi I (2012). Influence of age and gender on triglycerides-to-HDL-cholesterol ratio (TG/HDL ratio) and its association with adiposity index. Archives of Gerontology and Geriatrics.

